# Hepatocytes respond differently to major dietary *trans* fatty acid isomers, elaidic acid and *trans*-vaccenic acid

**DOI:** 10.1186/s12953-015-0084-3

**Published:** 2015-12-01

**Authors:** Toke P. Krogager, Lone Vendel Nielsen, Derya Kahveci, Thomas F. Dyrlund, Carsten Scavenius, Kristian W. Sanggaard, Jan J. Enghild

**Affiliations:** Department of Molecular Biology and Genetics and iNANO, Aarhus University, Gustav Wieds Vej 10C, 8000 Aarhus C, Denmark

**Keywords:** Cardiovascular disease, Lipid metabolism, Trans fatty acid, Proteomics, SILAC

## Abstract

**Background:**

It has been discussed if the adverse health effect associated with the ingestion of *trans* fatty acids correlates with the food source, as the composition of the isomers varies in different foods. We have investigated the hepatocellular responses to the predominant *trans* fatty acid isomers in industrially produced partially hydrogenated vegetable oils (elaidic acid) and products of ruminant origin (*trans*-vaccenic acid).

**Results:**

The responses of HepG2-SF cells exposed to 100 μM fatty acids during 7 days were examined. Elaidic acid decreased the cellular proliferation rate while *trans*-vaccenic acid had no effect. Analysis of cellular triacylglycerol fractions showed, that both *trans* fatty acids were metabolized by HepG2-SF cells, although elaidic acid, to a higher degree than *trans*-vaccenic, accumulated in the triacylglycerol fraction. Proteome analysis revealed that the overlap of differentially regulated proteins only contained four proteins, suggesting that the two *trans* fatty acid isomers affect the cells in different ways. The data are available via ProteomeXchange with identifier PXD000760.

**Conclusions:**

Our investigations revealed that the hepatocellular response to the two most abundant dietary positional C18:1 *trans* fatty acid isomers differ substantially. In addition, the results suggest that *trans*-vaccenic acid does not affect cholesterol metabolism adversely compared to elaidic acid.

**Electronic supplementary material:**

The online version of this article (doi:10.1186/s12953-015-0084-3) contains supplementary material, which is available to authorized users.

## Background

*Trans* fatty acids (TFA) in the human diet originate from industrial partial hydrogenation of vegetable oils (iTFA) and the naturally occurring TFA in milk and body fat of ruminants (rTFA). The intake of iTFA can reach 9 % of the total energy intake [1] whereas the intake of rTFA rarely exceeds 0.5 % [2]. Dietary iTFA causes increased ratios in plasma of total cholesterol and LDL-cholesterol to HDL-cholesterol and of apoB to apoA1, therefore, increasing the risk of cardiovascular disease (CVD) (reviewed in [3]). The impact of rTFA on human health has also been investigated, but the results have been inconclusive (reviewed in [4]). Most studies do not show a relationship between rTFA and CVD [5–7] although a few have indicated an inverse association [8, 9]. In contrast to this potential positive effect of rTFA in relation to CVD, a high concentration of rTFA in serum and erythrocytes results in an increased risk of breast cancer [10, 11].

The main iTFA isomer is the *trans*Δ9-C18:1 named elaidic acid (EA), whereas the main isomer of rTFA is the *trans*Δ11-C18:1 named *trans*-vaccenic acid (*trans*VA) (40–60 % of rTFA [12]). The *trans*VA originates from incomplete biohydrogenation of polyunsaturated fatty acids by microorganisms in the rumen of cows, goats and sheep [13]. Besides from being the major rTFA, *trans*VA is also a constituent of iTFA contributing up to 5 % of the fat in margarine and shortening. Additionally, EA is present in rTFA, where it may constitute up to 10 % of the total rTFA [12]. Consequently, both EA and *trans*VA intake may originate from several sources, complicating the evaluation of the impact of the individual isomer on human health. It is possible to enrich ruminant products for *trans*VA through feeding strategies, by increasing time on pasture and decreasing silage in cattle feed, but the effect on EA content has not been evaluated [14]. TFA isomer distribution and content in iTFA varies depending on starting oil, degree of hydrogenation, temperature and catalyst [15, 16]. Knowledge about responses to individual TFA isomers such as EA and *trans*VA can contribute in developing guidelines for the process of partial hydrogenation of vegetable oils and food formulations by the industry, but also in the context of feeding strategies for milk and meat production.

Despite indications that differential responses to positional isomers exist [17, 18], to our knowledge the TFA isomer specific effects in human have not been directly investigated through dietary intervention studies. Human clinical studies comparing health impact of diets enriched in dairy products with diets rich in hydrogenated vegetable oils exist [19–21]. However, the effect caused by the particular TFAs was difficult to ascertain because both diets contained TFA mixtures with isomers in different amount. In other studies, TFA replaced saturated fatty acids or polyunsaturated fatty acids to keep calorie-intake constant between experimental diets [22, 23]. The result of these studies is likely a mixed effect of excluding some fatty acids and including others, and it is difficult to determine the effects of particular dietary TFA isomers [4]. Only few studies have been conducted on cultures of human cells to assess isomer specific responses in regards to CVD risk. The studies have focused on inflammatory markers in endothelial cells [24] and monocytes [25], and the results indicate, that EA is the most detrimental of the fatty acids, while *trans*VA may even be anti-inflammatory.

Besides inflammatory conditions, a significant factor in development of CVD is the dysregulation of cholesterol and lipoprotein levels. The liver is responsible for the secretion and regulation of plasma proteins. In addition, ingested fatty acids are metabolized by the liver and together with cholesterol redistributed to other organs through lipoproteins. Thus, the hepatocyte responses to individual TFAs are essential for understanding the physiological effect of the intake of different TFAs.

Through proteomic, we have evaluated if human hepatocellular cell line, HepG2-SF, responses to supplemented EA and *trans*VA are comparable by reference to their *cis*-isomers, oleic acid (OA) and *cis*-vaccenic acid (*cis*VA). The cellular response to the TFAs EA and *trans*VA were compared and investigated by reference to their naturally occurring *cis*-isomers OA and *cis*VA. Due to cellular desaturation of *trans*VA into conjugated linoleic acid (CLA) this fatty acid was also included. The concentration of total free fatty acids in human blood has been reported to be as high as 435 ± 11 μM and that of a single free fatty acid, such as oleic acid, can be as high as 158 ± 5 μM [26]. Previous studies of HepG2 cells showed physiological relevant responses to free fatty acids (ranging from 50 to 1000 μM) and further HepG2 cells expressed and secreted a broad range of known plasma proteins important to lipid metabolism [27–33]. The lower concentration range of free fatty acids (50–200 μM) only had a small effect on the cells [29, 31], whereas the free fatty acids were cytotoxic at higher concentrations [28]. These previous studies using HepG2 cells mainly investigated OA, EA and palmitic acid in relations to changes in cholesterol and lipoproteins levels. We used 100 μM free fatty acid in complex with human serum albumin, which mimics the natural transportation of free fatty acids in the blood when fatty acids are not transported as lipoproteins [32]. Further, we used the commercial available HepG2-SF cell line adapted to growth in serum free media (commercial available as SynQ), which contains a minimal fatty acid formulation (see Methods), this gives us crucial control of the presence of fatty acids in the cell culture media supplemented with the experimental fatty acids.

We used Stable Isotope Labeling by Amino acids in Cell culture (SILAC) to investigate both cellular and secreted proteins from HepG2-SF cells in two separate triplex setups where cells were supplemented with *trans*VA, *cis*VA or EA and *trans*VA, OA or CLA. This was chosen because of the limitation of multiplexing more than three groups with SILAC. Further, Difference in gel electrophoresis (DIGE) was used to investigate the secretome from HepG2-SF cells supplemented with EA, OA or *cis*VA. Special emphasis has been on analyzing the secretome of EA and *trans*VA supplemented cells, as changes in particular plasma protein levels are essential in the progression of CVD.

## Results

### Viability of HepG2-SF cells in fatty acid supplemented medium

**Fig. 1:**
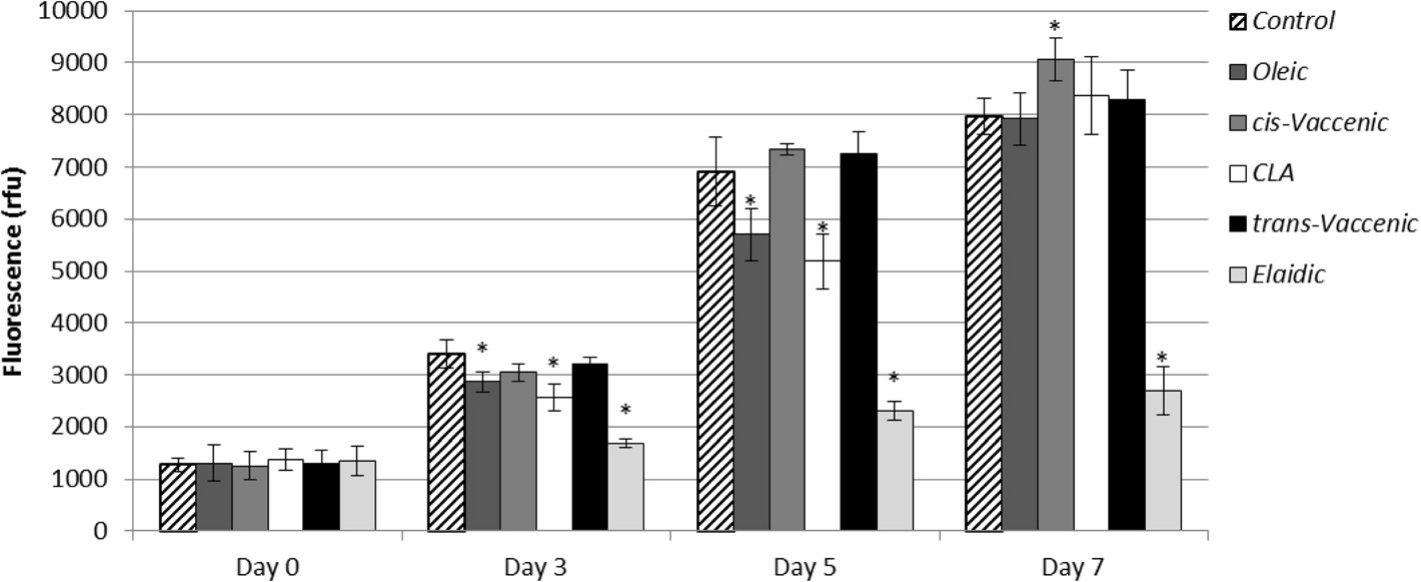
Viability and proliferation of HepG2-SF cells in fatty acid supplemented medium. Viability of HepG2-SF cells in medium supplemented with 100 μM OA, *cis*VA, CLA, *trans*VA, EA, or no fatty acid (*Control*) was investigated using CyQuant proliferation assay. The measured fluorescence (y-axis) corresponds to live cell numbers. During a seven days period *cis*VA, t*rans-*VA*,* and *Control* do not markedly differ in proliferation rate, whereas EA*,* though still viable, appear to be compromised on their proliferation. *significantly different from *Control* at *p* < 0.05

HepG2-SF cell viability in 100 μM fatty acid supplemented medium was analyzed using CyQuant NF proliferation assay, as some C18 unsaturated fatty acids may be cytotoxic for HepG2 cells at or above 100 μM [34]. During 7 days exposure, HepG2-SF cells were found viable in the presence of 100 μM of all the fatty acids tested (Fig. 1). Proliferation did not significantly differ between *cis*VA and *trans*VA and these did not differ markedly from Control (no fatty acid supplementation) either. Cells supplemented with CLA and OA did not significantly differ from Control in cell numbers on day 7, however, CLA and OA supplemented cells showed lower proliferation rates at the beginning of the experimental period. Supplementation with EA causes a considerable decrease in proliferation rate as compared to the other experimental groups, which suggests that EA directly or indirectly inhibit proliferation.

### Fatty acid esterification into TG of HepG2-SF cells

**Fig. 2:**
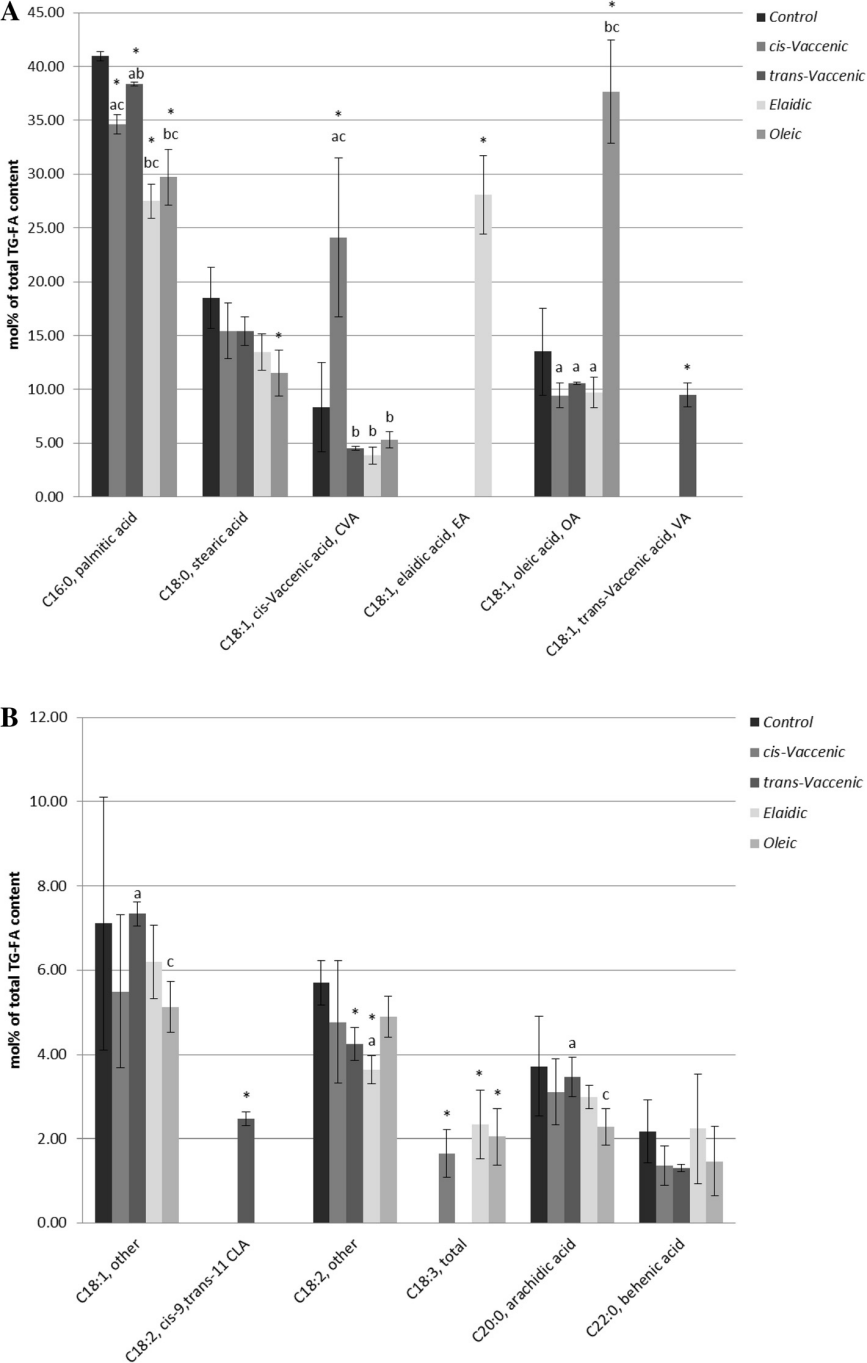
The fatty acid composition of HepG2-SF TG after fatty acid supplementation. HepG2-SF TG composition was analyzed by gas liquid chromatography after 7 days incubation in fatty acid supplemented medium (100 μM). Panel **a** and **b** are high and low abundant fatty acids present in the TG, respectively. Content of individual fatty acids (x-axis), analyzed as extracted FAME, are averaged over three biological replicas and displayed as % of total TG derived FAME measured (y-axis). The supplemented TFAs esterify at different levels into HepG2-SF TG and EA is found at approximately three times the level of *trans*VA. The analysis shows that the supplemented fatty acids are taken up and metabolized by HepG2-SF cells. In addition, it demonstrates that EA is accumulating in TG when compared to the other experimental groups. *significantly different from *Control* at *p* < 0.05. Fatty acids significantly differing between experimental groups at *p* < 0.05 are marked with: a) different from OA*,* b) different from *cis*VA and c) different from *trans*VA

To test if the supplemented fatty acids were taken up by the HepG2-SF and incorporated into TG, the fatty acid composition of FAME derived from the HepG2-SF TG fraction was analyzed by gas–liquid chromatography (Fig. 2 and Additional file 1: Data S1). The result showed that HepG2-SF cells take up the different supplemented fatty acids and esterify them into their TG. Eukaryotes are not able to make the fatty acid double bond of the *trans* geometry, and so Control and cells supplemented with *cis*VA do not contain TFA. Upon *cis*VA supplementation the content of this fatty acid in cell TG increase from (8.3 ± 4.2) % to (24.1 ± 7.4) % (i.e. the supplemented *cis*VA corresponds to 15.8 % ± 8.5 of total TG derived FAME). Supplementing HepG2-SF cells for 7 days with *trans*VA, this fatty acid is present at 9.5 % ± 1.1 of the TG (Fig. 2a). The relative amounts of the supplemented geometrical isomers *trans*VA and *cis*VA added to HepG2-SF TG are thus comparable (9.5 % ± 1.1 and 15.8 % ± 8.5). This is in contrast to the relative amounts of the positional *trans*-isomers EA and *trans*VA, which are very different, with EA present at approximately three times the concentration of *trans*VA (28.1 % ± 3.6 versus 9.5 % ± 1.1). In humans, *trans*VA is desaturated into CLA with a conversion rate estimated to be 19 % [35]. We observed 2.47 % ± 0.16 CLA, when supplementing with *trans*VA, corresponding to 21 % of the combined *trans*VA and CLA (Fig. 2b), thus reflecting the estimated human conversion rate. Palmitic acid is the measured non-supplemented fatty acid that differed the most in amount between experimental groups, and is lower when cells were supplemented with EA and OA than compared to both supplementation with *trans*VA or *cis*VA (*p* < 0.05) and the largest difference is observed between *trans*VA and EA supplementation*.* In conclusion, HepG2-SF cells take up and metabolize all the supplemented fatty acids. However, after 7 days of supplementation, the measured amounts of EA and *trans*VA isomers in TG differed between experimental groups, indicating a differential lipid metabolic response to the TFA isomers.

### Identification and quantitation of proteins differentially regulated by *trans*VA, EA and *cis*VA using SILAC

SILAC was applied to investigate the cellular proteomic response of the predominant rTFA and iTFA , *trans*VA and EA respectively, and compare their response to the cisVA Both the secreted and the intracellular proteins were investigated. A total of 3074 proteins were identified and of these 538 were quantifiable across all three groups. In the *trans-trans* comparison EA/VA 25 proteins were significantly regulated with a fold > 1.3. In the *trans-cis* comparison *trans*VA/*cis*VA 20 proteins were significantly regulated with a fold > 1.3, and in EA/*cis*VA 24 proteins were significantly regulated with fold > 1.3. A total of 47 differentially regulated proteins were found (*p* < 0.01, fold > 1.3, Table 1 and Additional file 2: Data S2). Functional clustering analysis showed that the most significant functions represented by the regulated proteins were lipid and cholesterol metabolic processes including specifically cholesterol biosynthetic process. Except for apoA1, the regulated proteins involved in cholesterol synthesis are specifically up-regulated by EA and not *trans*VA, demonstrating that this response is isomer specific. Eight of the 17 proteins categorized in lipid metabolism process are up-regulated by EA when compared to both *trans*VA and *cis*VA and only 3 non-lipid metabolism proteins (tricarboxylate transport protein–mitochondrial, membrane-associated progesterone receptor component 1, and filamin-B) are regulated in these comparisons.

**Table 1:**
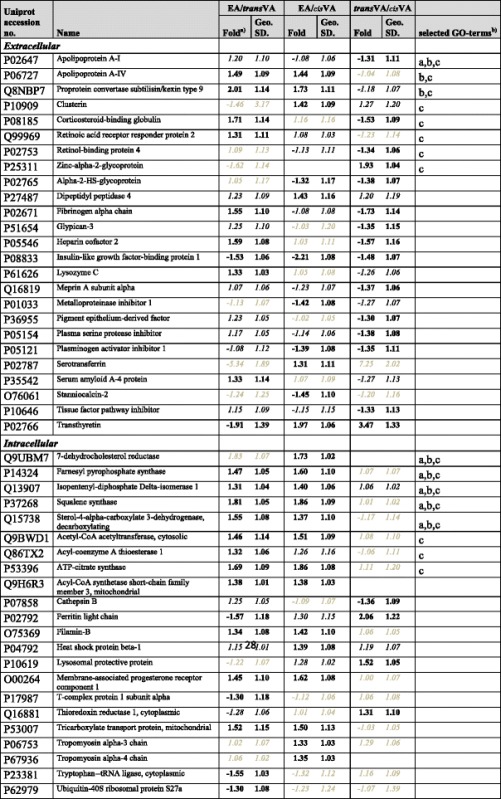
Differentially regulated proteins between *trans-Vaccenic, cis-Vaccenic and Elaidic* revealed by SILAC

Proteins are reported with Uniprot accession number, Name, Fold regulation in individual comparisons, the geometric standard deviation (Geo.SD.) and selected GO-terms

a) black bold: significant regulatory data (*p* < 0.01) with fold > 1.3, black italic: significant regulatory data (*p* < 0.01) with fold < 1.3, grey italic: not significant regulatory data (*p* > 0.01)

b) a: cholesterol biosynthetic process (GO:0006695), b: cholesterol metabolic process (GO:0008203), and c: lipid metabolic process (GO:0006629)

Corticosteroid binding protein, classified in lipid metabolism, was down-regulated upon *trans*VA-supplementation in both *trans*VA/EA and *trans*VA/*cis*VA. Heparin cofactor 2 and fibrinogen alpha chain are non-lipid metabolism proteins regulated by *trans*VA-supplementation. They were both down-regulated. Three proteins were down-regulated by both TFAs when compared to *cis*VA*,* these were plasminogen activator inhibitor 1 (PAI1), alpha-2-HS-glycoprotein (AHSG) and insulin-like growth factor-binding protein 1 (IGFBP1). Transthyretin (TTR) is up-regulated in both *trans*VA/*cis*VA and EA/*cis*VA.

The SILAC analysis showed that the overlap of differentially regulated proteins between the comparisons EA/*cis*VA and *trans*VA/*cis*VA is rather small, comprising 16 % and 20 %, respectively, of regulated proteins at fold > 1.3, *p* < 0.01. This suggests that cellular responses to the two major dietary TFAs are rather different. However, a shared TFA-induced response was observed for PAI1, AHSG, IGFBP1, and TTR, as mentioned above.

### Identification and quantitation of secreted proteins differentially regulated by *trans*VA, EA and *cis*VA using DIGE

To further investigate the effects of TFA isomers on plasma protein levels and to gain more information on differentially regulated secreted proteins, the experimental medium from fatty acid supplementations of HepG2-SF cells were analyzed by DIGE. Approximately 2000 spots were detected per gel and 1000 aligned across all analytical gels. Using the criteria of 1.3 fold regulation at *p*-value < 0.05 the numbers of differentially regulated spots were 44 in EA/*trans*VA and 5 in *trans*VA/*cis*VA. From the 44 differentially regulated spots in the *trans-trans* comparison EA/*trans*VA, 12 unique proteins (excl. human serum albumin) were identified, and from the 5 differentially regulated spots in the *trans-cis* comparison *trans*VA/*cis*VA, 3 unique proteins were identified (Table 2 and Additional file 3: Data S3). EA, compared to *trans*VA, induced up-regulation of apoA1 and apoE, which are apolipoproteins of HDL and VLDL respectively, and down-regulation of AHSG, which inversely correlate with CVD risk [36] and is suggested as a biomarker for TFA intake [37]. ApoA4 was up-regulated in both EA/*trans*VA and *trans*VA/*cis*VA. Four spots, upregulated by *trans*VA when compared to EA, were identified as complement C3 with migration in the 2D gel estimated at a pI a little below 5 and a mass of approximately 40 kDa (Table 2, Additional file 3: data S3 and Additional file 4: Figure S1). The peptides, derived from the spots and identifying the proteins as complement C3, all locate in the c-terminal part of C3 corresponding to the C3c alpha’ chain fragment 2. This also fits the mass and pI observed, thus indicates increased complement C3 activation by *trans*VA. The DIGE results thus support and supplement the SILAC results, as will be discussed, which further underline the indication that the major iTFA (EA) and the major rTFA (*trans*VA) promote relatively different hepatocellular responses.

**Table 2:** Differentially regulated secreted proteins revealed by DIGE and identified by mass spectrometry

			EA/*trans*VA		*trans*VA/*cis*VA	
Protein name	Uniprot name	spot no	Av. Ratio	*p*-value	Av. Ratio	*p*-value
14-3-3 protein zeta/delta	1433Z/P63104	2180			−1.43	3.0E-02
Aldose 1-epimerase	GALM/Q96C23	1832	2	2.5E-03		
Alpha-1-antitrypsin	A1AT/P01009	1330	1.79	3.6E-04		
Alpha-1-antitrypsin		1342	1.96	3.9E-03		
Alpha-2-HS-glycoprotein	FETUA/P02765	1400	−1.51	3.4E-02		
Apolipoprotein A1	APOA1/P02647	2333	1.57	2.0E-04		
Apolipoprotein A4	APOA4/P06727	1737	1.57	7.3E-03	1.39	4.8E-02
Apolipoprotein A4		1741	1.7	1.7E-04	1.4	2.8E-03
Apolipoprotein A4		1743	1.43	2.3E-02		
Apolipoprotein E	APOE/P02649	2030	1.39	5.9E-03		
Carboxypeptidase E	CBPE/P16870	1417	−1.78	1.3E-03		
Chloride intracellular channel protein1	CLIC1/O00299	2106	1.75	1.9E-03		
Complement C3	CO3/P01024	1675	−1.41	3.1E-02		
Complement C3		1706	−1.51	4.6E-03		
Complement C3		1708	−1.51	4.6E-03		
Complement C3		1710	−1.51	8.8E-04		
Complement factor B	CFAB/P00751	726	−1.32	2.5E-03		
Endoplasmin	ENPL/P14625	737	−1.51	4.8E-02		
Proteasome subunit alpha type-5	PSA5/P28066	2203			−1.37	4.7E-02
Protein AMBP	AMBP/P02760	2042	1.62	2.2E-03		
Serotransferrin	TRFE/P02787	1005	1.56	8.0E-04		
Serotransferrin		1007	2.02	2.7E-04		
Serotransferrin		1033	1.65	1.5E-04		
Serotransferrin		1765	1.43	1.1E-02		

The secretome of hepatocytes supplemented with *trans*VA, EA, or *cis*VA were analyzed by DIGE and subsequently the regulated proteins were identified by LC-MS/MS analyses. In the table proteins are reported with protein name, Uniprot name/accession number, 2D gel spot number (spot no), Average ratio (Av.ratio), and *p*-value for individual comparisons

### Identification and quantitation of proteins differentially regulated by *trans*VA, OA and CLA using SILAC

OA is the most abundant of naturally occurring C18:1 *cis* fatty acids in food stuff, and it was included in a separate SILAC analysis comparing OA-supplemented cells to *trans*VA*,* to test if the *trans*VA induced response, observed in the *trans*VA-*cis*VA-EA comparison described above, could also be confirmed with reference to OA. CLA was included in this second SILAC setup, as mammalian cells are able to further desaturate *trans*VA to yield CLA. The cellular response to 100 μM CLA was investigated to assess if the *trans*VA to CLA conversion contributes to the observed *trans*VA response.

A total of 3191 proteins were identified in this second SILAC triplex setup and of these 15 were differentially regulated in the *trans*VA/OA comparison within the fold 1.3 criteria set, *p* < 0.01. For CLA/*trans*VA and CLA/OA the numbers were 26 and 39, respectively (Additional file 5: Data S4). Five of the 15 proteins significantly regulated in *trans*VA/OA were also regulated in the first SILAC *trans*VA-*cis*VA-EA triplex. The down-regulation of AHSG, fibrinogen alpha chain and corticosteroid binding globulin was confirmed in *trans*VA/OA together with down-regulation of glypican 3 and stanniocalcin 2.

If the cellular response to *trans*VA was in part mediated by its conversion to CLA, supplementation with 100 μM CLA would be expected to enhance the response observed during supplementation with *trans*VA*.* Ten of the 26 proteins significantly regulated in the CLA/*trans*VA comparison were also regulated in the first SILAC *trans*VA-*cis*VA-EA triplex. Eight of these were in CLA/VA regulated in the opposite direction as in the *trans*VA/*cis*VA comparison. This demonstrates that the *trans*VA induced response is not significantly contributed by the presence of CLA, and furthermore the data suggests that the *cis*-desaturation on the ∆9 position in CLA may neutralize some of the effects of the ∆11 *trans*-double bond.

## Discussion

Investigations of adverse health effects related to TFA intake have not focused on the contribution of the individual positional TFA isomers. We have in earlier studies investigated the differential response of human hepatocytes, HepG2-SF, to the two geometric ∆^9^C18:1 isomers OA (*cis*∆^9^C18:1) and EA (*trans*∆^9^C18:1). The studies revealed substantial differences in abundance of proteins involved in cholesterol synthesis and lipid metabolism in general and furthermore indicated remodeling of the phospholipids caused by the EA supplementation of the cells [37, 38]. In the present study, we investigate if the hepatocellular responses to the two most abundant positional C18:1 TFA isomers in the human diet, EA and *trans*VA, are comparable. The *cis*VA was included to test if also the ∆^11^C18:1 geometrical isomers showed the same affect on HepG2-SF cells, as previously shown for the ∆^9^C18:1 geometrical isomers.

### Supplemented *trans* fatty acids esterify at different amounts into the TG of liver cells

To our knowledge, TFA induced hepatic responses have not earlier been investigated in human cell cultures, although a study using rat hepatocytes showed that short-term (2 h) exposure to EA or *trans*VA causes equal incorporation of the TFAs into TG [39]. Contrary to the results in rats, we showed, by the analyses of FAME derived from HepG2-SF TG, that after 7 days supplementation with EA or *trans*VA that the TG fraction contained more than double the amount of EA to that of *trans*VA. Upon supplementation, *trans*VA and *cis*VA esterified to similar extend into the TG fraction. TG acts as a cellular storage site for fatty acids, which can be used for energy production through β-oxidation in mitochondria and peroxisomes. In rat the β-oxidation rate of EA has been shown to be 30 % lower than for both *trans*VA and *cis*VA [40] because a β-oxidation intermediate of EA slows β-oxidation progress [41]. If this occurs in human cells, EA may thus be accumulating in TG as β-oxidation slows down and this may be the reason for our observation of different relative amounts of TFAs in HepG2-SF TG. However, the total amounts of TG per cell in each experimental group were not determined in the present study. Consequently, it cannot be concluded if EA-containing TGs are added to the TG pool or if EA substitutes for other fatty acids, for example palmitic acid, keeping total TG at a constant amount.

### EA and *trans*VA affect lipid metabolism-related proteins differently

The cellular proteomic responses to EA, *trans*VA and *cis*VA were analyzed using SILAC and DIGE. The SILAC analysis identified 47 differentially regulated proteins in EA/*trans*VA, *trans*VA/*cis*VA and/or EA/*cis*VA (Table 1) and functional cluster analysis showed that the main perturbed cellular functions are lipid and cholesterol metabolism. In a previous study, comparing the HepG2-SF responses to EA and OA, we found that the major part of proteins associated with cholesterol synthesis were up-regulated in the TFA treated cells [38]. Moreover, in rat hepatocytes EA supplementation caused an increased cholesterol efflux as compared to both *trans*VA and *cis*VA [39]. In the present study, we find several of the proteins involved in lipid metabolism, and especially cholesterol synthesis, to be up-regulated by EA, when compared to both *trans*VA and *cis*VA. It supports the findings in rats and suggests that the increased cholesterol efflux is in part a result of increased synthesis. Also, Proprotein convertase subtilisin/kexin type 9 (PCSK9) was up-regulated in both EA/*trans*VA and EA/*cis*VA but unchanged in *trans*VA/*cis*VA. This protein can cause degradation of LDL-receptor [42] and thereby possibly decrease the LDL-cholesterol uptake by the cell contributing to extracellular cholesterol accumulation. DIGE was performed on the secreted proteins from HepG2-SF cells, because it has been suggested that TFA may influence CVD risk by altering secretion of plasma proteins from the liver. Our DIGE data shows that apoA1, which is the major apolipoprotein of HDL, is up-regulated by EA when compared to *trans*VA and unchanged in *trans*VA/*cis*VA (Table 2). In SILAC the regulatory data for apoA1 is 1.2 fold for EA/VA and −1.3 fold for *trans*VA/*cis*VA. Cholesteryl ester transfer protein is responsible for cholesteryl ester transfer from HDL to LDL and its activity is increased by TFA [43]. If ApoA1 is up-regulated by EA, this may cause an increased extracellular capacity for cholesterol, and with increased CETP activity this may aid cholesterol accumulation in LDL.

### *Trans* fatty acid specific responses

The SILAC analysis revealed four proteins that were significantly regulated in the same direction, but not necessarily to the same extent, by both TFAs when compared to *cis*VA. These included TTR, which is up-regulated, and PAI1, IGFBP1 and AHSG, which are down-regulated. We have in a previous biomarker study compared EA with OA and among other candidates found TTR, PAI1, IGFBP1 and AHSG as potential biomarkers for TFA-intake [37]. The present study supports that the regulation of these proteins are TFA specific. Decreased level of metalloproteinase inhibitor 1 (TIMP1) was also suggested as a potential biomarker for EA intake [37], and this protein was down-regulated in EA/*cis*VA (−1.42) and just below the fold cut-off in *trans*VA/*cis*VA (−1.27), also indicating this to be a TFA-specific response.

In the previous biomarker study PCSK9, ApoA4, tissue factor pathway inhibitor (TFPI), AHSG, and serotransferrin were also shown to be potential biomarkers for EA supplementation, when compared to OA [37]. PCSK9, ApoA4 and serotransferrin were up-regulated, TFPI and AHSG down-regulated. In the present investigations, SILAC analysis confirmed that PCSK9 and ApoA4 were up-regulated and potential EA biomarkers. TFPI was down-regulated by *trans*VA when compared *to cis-*VA (−1.33), and the fold down-regulation in EA/*cis*VA was 1.15. Serotransferrin was up-regulated in EA/*cis*VA (1.31), but lacked significance in regulation in the other comparisons. In the DIGE characterization of the proteins secreted by HepG2-SF cells, ApoA4 and serotransferrin were up-regulated (on average 1.57 and 1.67, respectively) and AHSG down-regulated (−1.51) in the EA/VA comparison (Table 2), confirming the observed changes in the EA/OA comparison [37] underlining that these are potential biomarkers for EA intake. However, ApoA4 was also up-regulated in the DIGE *trans*VA/*cis*VA comparison (1.4), which shows that this response is not EA-specific though more pronounced by EA than by *trans*VA supplementation. Noteworthy, all the regulated and discussed proteins (PCSK9, AHSG, ApoA4, TIMP1, TFPI, PAI1 and IGFBP1) have previously been mentioned in the context of CVD risk [36, 37, 44–47].

However, 18 of the 26 proteins differentially regulated in CLA/*trans*VA categorize with the GO-term: Response to stress, all up-regulated by CLA, and seven of these are part of the acute inflammatory response. This may indicate that the high concentration of CLA is stressful or even toxic for HepG2-SF cells, though not decreasing viability after a seven days period. A blood concentration of 100 μM CLA is not easily reached through diet, and though interesting it is beyond the scope of this paper to further discuss the cellular response to CLA.

### The complement system is affected by TFA

Artherosclerosis is considered an inflammatory condition [48]. It has been suggested that dietary TFA induces vascular inflammation and effects on several markers of endothelial cell dysfunction and inflammatory responses have been documented [24, 49, 50]. Though a TFA, *trans*VA did not induce this effect in endothelial cells and may even protect against vascular inflammation measured as decreased T-helper cell cytokine production [24, 25]. Part of the vascular inflammatory response is the activation of the complement system, and a key protein is complement factor C3 (CO3). Blood CO3 is a predictor of myocardial infarction [51] and correlates positively with obesity and coronary artery disease [52]. In the DIGE study we identify CO3 in 4 spots, up-regulated by *trans*VA when compared to EA and unchanged when compared to *cis*VA (Table 2, Additional file 3: Data S3 and Additional file 4: Figure S1)*.* These spots correspond to the most C-terminal part of CO3, the C3c alpha chain fragment 2. By SILAC it is observed that the overall CO3 level is significantly increased by EA compared to *trans*VA (1.27 fold), and up-regulation in *cis*VA/VA is also indicated (Additional file 2: Data S2). The regulatory data for CO3 suggests that supplementation with *trans*VA gives the lowest overall level of CO3, but the highest level of CO3 activation relative to total amount. Complement factor B is also an important protein in the complement system, which upon binding to CO3 b fragment interacts with and activates complement factor D, which in turn cleaves factor B [53]. The cleaved form of factor B forms a complex with the CO3 b fragment in the formation of C3 convertase, which further accelerate the cleavage of CO3. Complement factor B was significantly up-regulated 1.2 fold in SILAC in both EA/VA and *cis*VA/*trans*VA i.e. down-regulated by *trans*VA (Additional file 2: Data S2). In DIGE judging by mass, pI, spot pattern, and identification by MS/MS, the full-length complement factor B was observed as a train of spots, with one spot up-regulated 1.42 fold and another down-regulated 1.32 fold in EA/VA. This observation in DIGE suggests a change in the glycosylation pattern, with an EA induced deglycosylation of factor B. Deglycosylation at Asn260 of factor B causes increased affinity for the CO3 b fragment [54] and deglycosylation at Asn353 is suggested to increase cleavage by factor D and thus deglycosylation at both sites can increase formation of the C3 convertase [55]. Whether the observed change in spot pattern on our 2D gels is due to deglycosylation at either of the mentioned sites, with implication for complement activation due to EA, requires further investigations.

Some proteins categorized in the acute inflammatory/acute phase response are also regulated by the TFAs investigated, though not clearly confirming or rejecting an acute phase response. These include the positive acute phase proteins: fibrinogen alpha chain (SILAC: EA/VA 1.55 fold, *trans*VA/*cis*VA −1.73 fold), PAI1 (SILAC: EA/*cis*VA −1.39, *trans*VA/*cis*VA −1.35), serum amyloid A-4 (SILAC: EA/*trans*VA 1.33, *trans*VA/*cis*VA −1.27) and ferritin light chain (SILAC: EA/*trans*VA −1.57, *trans*VA/*cis*VA 2.06), and the negative acute phase proteins: AHSG (SILAC: EA/*cis*VA −1.32, *trans*VA/*cis*VA −1.38; DIGE EA/VA −1.51), serotransferrin (SILAC: EA/*cis*VA 1.31; DIGE EA/*trans*VA 1.67) and TTR (SILAC: EA/*cis*VA 1.97, *trans*VA/*cis*VA 3.47).

### Regulators of blood coagulation are affected by TFA

By SILAC fibrinogen alpha chain was the only protein to be significantly regulated specifically by *trans*VA when compared to both EA and *cis*VA (−1.55 and −1.73 fold regulation, respectively). Though not significant at *p* < 0.01 both fibrinogen beta and gamma chain also showed approximately 1.6 fold down-regulation by *trans*VA supplementation (Additional file 2: Data S2). PAI1 was decreased by both EA and *trans*VA. Increased plasma fibrinogen and PAI1 levels correlates with increased thrombosis risk [44, 56], and thus *trans*VA may have a potential beneficial influence on the level of these proteins in regards of CVD risk. However, the observed decrease in level of fibrinogen could also indicate a *trans*VA-induced shift towards coagulation of the equilibrium between coagulation and fibrinolysis, resulting in consumption of fibrinogen. The possibility of increased coagulation is supported by the observation that both TFPI and heparin cofactor 2 are also decreased by *trans*VA-supplementation. TFPI is a major inhibitor of the extrinsic coagulation pathway through its interaction with Factor Xa and tissue factor-factor VIIa complex [57, 58], and heparin cofactor 2 inhibits thrombin action [59], thus low level of both may promote thrombophilia. Whether *trans*VA promotes or protects against thrombophilia is thus difficult to conclude from the present experiments, however it clearly affects levels of regulators of blood coagulation.

## Conclusion

It has previously been established that iTFAs correlate with CVD risk [3]. However, a diet very high in rTFA also negatively affects CVD risk factors [20]. It has therefore previously been discussed if *trans*VA and EA in food have different effects on human health, or whether it is merely a matter of the total dietary TFA amount consumed [60]. Our study shows that partially differential hepatocellular responses to the two C18:1 TFA isomers *trans*VA and EA occurs. EA specifically induces an increase in proteins involved in cholesterol synthesis and cholesterol transport, a response not shared with *trans*VA. The results of this investigation suggest that EA has an adverse effect on cholesterol metabolism that could be unique to the main TFA isomers found in foodstuff and explain its stronger correlation with CVD risk than for *trans*VA, but this requires more investigations in animal models and ultimately in clinical intervention studies.

## Methods

### Materials

*Cis*-vaccenic acid (> 97 % purity) and Conjugated linoleic acid (> 96 % purity) were purchased from Cayman Chemicals. ^13^C_6_Arginine and ^13^C_6_^15^N_4_Arginine (both 99 % purity) were from Cambridge isotope laboratories (Andover, MA, USA). Elaidic acid, Oleic acid, *trans*-vaccenic acid , L-Lysine and Arginine (all 99 % purity), Anhydrous dimethyl formamid (99.5 % purity), iodoacetamide, tetra-methyl-ethylene-diamine, L-Glutamine, Human serum albumin and RPMI-1640 without Arginine, Lysine and Leucine were purchased from Sigma. Regular SynQ serum substitute and SynQ without Arginine and Lysine was from Cell Culture Service, Hamburg Germany. All reagents for DIGE were PlusOne quality from GE Healthcare. Also CyDye DIGE fluors, IPG running buffers and PD10 columns were purchased from GE Healthcare. Penicillin, Streptomycin, regular RPMI-1640 and CyQuant NF proliferation assay kit were from Invitrogen.

### Cell culture

HepG2-SF cells (HepG2 cells optimized for serum free growth, Cell Culture Service, Hamburg, Germany) were propagated in 75 cm^2^ Nunc^™^ culture flasks in serum free medium composed of RPMI-1640, 10 % SynQ Serum substitute (Contains: Oleic acid, 3.5 μM; Linoleic acid, 500 ng/mL; Thioic acid, 250 ng/mL), 4 mM L-glutamine, 20 U/mL penicillin and 20 μg/mL streptomycin, which was changed every third day. Cells were maintained at 37 °C in a 5 % CO_2_ humidified incubator and subcultured every two weeks. For preparation of fatty acid supplemented medium, fatty acid in a 2:1 molar complex with human serum albumin [61] was adjusted to a final concentration of 100 μM in serum free medium.

### Assessment of HepG2-SF growth in fatty acid supplemented medium by CyQuant proliferation assay

In a 96 well (low fluorescence) microtiter-plate 5,500 cells were seeded per well and allowed to attach for 24 h before 300 μl 100 μM fatty acid supplemented regular medium was added (4 replicas for each experimental group). On day 0, 3, 5 and 7 after addition of supplemented media proliferation was assessed by adding 100 μl CyQuant dye solution per well, and after 30 min incubation at 37 °C fluorescence was measured using a FLUOstar Omega (BMG Labtech) until a plateau was reached (excitation 480 nm, 520 nm emission, 3 h). For comparison of experimental groups with *Control* equality of variance were tested (F-test) and appropriate two-tailed t-tests were performed.

### Measurement of fatty acids esterified into triacylglycerols (TG) by gas–liquid chromatography

For each experimental group 3 replicas of three million cells each, incubated 7 days in fatty acid supplemented serum free medium as described above, were washed 2 times in sucrose buffer (250 mM sucrose, 50 mM KCl, 2 mM MgCl_2_, 20 mM Tris–HCl, pH 7.6) and scraped off the petridish in 1 ml sucrose buffer. Cells were centrifuged for 3 min at 180 g and the supernatant was discarded. Pellets were resuspended in methanol, vortexed and transferred to glass tubes with Teflon lined caps. Lipids were extracted with methyl-tert-butyl-ether according to [62] and subjected to thin layer chromatography on 20 × 20 cm silica gel 60 plates with glass backing (Merck) using a two-buffer system (buffer 1, 30 min: chloroform:methanol:acetic acid:water-50:30:8:3, buffer 2, 45 min: heptane:diethyl ether:acetic acid – 70:30:2). Plates were dried after the development and lipids were visualized by placing the plates in an iodine chamber, the TG bands were marked and scraped off the plate when iodine was evaporated. TG were extracted from silica with chloroform:methanol (2:1) and dried. Fatty acid methyl esters (FAME) were generated from the TG using BF_3_. First samples were mixed with 1 ml methanolic NaOH (0.5 M) and heated for 5 min at 80 °C. After cooling to room temperature 1 ml of BF_3_-methanol (13–15 %) was added and again samples were heated at 80 °C for 2 min and cooled rapidly to room temperature. To improve FAME extraction to the organic phase, 0.2 ml of a salt solution containing NaCl_2_ (370 mg/ml) and K_2_CO_4_ (1.5 mg/ml) in water was added followed by 1 ml of heptane. After 5 min centrifugation at 4000 rpm the upper organic phase was isolated and dried over anhydrous Na_2_SO_4_ before application to gas–liquid chromatography. The FAME were analyzed on a Thermo Trace GC Ultra (Thermo Scientific, USA) equipped with auto-sampler, flame ionization detector and a Supelco SLB-IL100 column (60 m x 0.25 mm x 0.2 μm film thickness, Sigma-Aldrich). Helium was used as a carrier gas with a flow rate of 1 ml/min. An isothermal program at 180 °C for 25 min was applied. The injector and detector temperatures were set at 200 °C and 220 °C, respectively. Five μl samples with a split flow of 10:1 were injected. Using Xcalibur software fatty acids were identified by comparing their retention times to standard mixtures and those eluting between 11.5 min and 23.5 min are expressed as mol % based on an internal standard (methyl heptadecanoate).

### Incubation of HepG2-SF cells in fatty acid supplemented media for Stable Isotope Labeling by Amino acids in Cell culture (SILAC)

Serum free medium was prepared from RPMI-1640 without Arginine, Lysine and Leucine and SynQ without Arginine and Lysine. The medium was then supplemented with Leucine and Lysine to a final concentration of 0.38 mM and 1.02 mM, respectively. From this medium three different SILAC growth media were prepared containing 1.15 mM Arginine, ^13^C_6_Arginine or ^13^C_6_^15^N_4_Arginine. To facilitate full incorporation of ^13^C_6_Arginine and ^13^C_6_^15^N_4_Arginine the cells were cultivated as described above for 5 population doublings with medium change every third day. The level of incorporation was tested by MALDI-MS prior to incubations with fatty acids in SILAC media. For preparation of fatty acid supplemented medium fatty acid in a 2:1 complex with human serum albumin [61] was adjusted to a final concentration of 100 μM in SILAC serum free medium. Two SILAC triplex setups were made one using *trans*VA (Arginine), EA (^13^C_6_Arginine) and *cis*VA (^13^C_6_^15^N_4_Arginine), the other using *trans*VA (Arginine), OA (^13^C_6_Arginine) and CLA (^13^C_6_^15^N_4_Arginine).

For each experimental group 75 cm^2^ culture flasks containing 90 % confluent cells were trypsinized and seeded into four 6 cm petri dishes (= four replicas/experimental group). Cells were allowed to attach for 24 h before 3 mL fatty acid supplemented medium was added. The medium was changed on day two, four and six. On day 7 medium was aspirated and filtered (Sarstedt syringe filters, 0.22 μm) and kept on ice until further processing as described below. Cells were trypsinized, washed three times in cold PBS and lysed in lysis buffer (1 % NP-40, 150 mM NaCl, 50 mM Tris, 0.1 % SDS, 1 mM PMSF, 25 μM E64, 2 mM 1.10 Phenanthroline, pH 7,4) by sonication on ice. Lysate was centrifuged (16000 g, 4 °C) and supernatant collected and kept on ice.

### SILAC sample preparation

Cell medium was depleted for albumin by affinity chromatography on a column with a recombinant albumin binding domain from Streptococcal protein G [63]. The flow through was collected and the column regenerated using 20 mM Na-citrate, 150 mM NaCl, pH 2.5 before application of next sample. Between experimental groups the column was cleaned using 30 % isopropanol, 2 M NaCl. Protein concentration in depleted samples were determined using Bradford assay kit [64]. The 4 biological replicas in each group were pooled based on protein concentration and then the pools from different fatty acid incubations were mixed in a 1:1:1 ratio. The protein concentration in cell lysates were determined by 2D-Quant kit (GE Healthcare) and mixed as described for cell medium samples. Proteins from medium and lysates were separated in individual lanes by SDS-PAGE [65] and each lane was cut into bands. Proteins in the bands were in-gel digested using trypsin [66] and subsequently the resulting peptides were desalted on C18 stage tips (Proxeon).

### LC-MS/MS of SILAC samples

The peptides were separated on an Easy-nLC II HPLC system (Thermo Scientific) equipped with a trap column (ReproSil-Pur C18-AQ (5 μm, 2 cm x 100 μm I.D., Thermo Scientific) and an analytical column (ReproSil-Pur C18-AQ column, 3 μm, 10 cm x 75 μm I.D., Thermo Scientific) in-line to a NanoSpray III source (AB Sciex) connected to a TripleTOF 5600 mass spectrometer (AB Sciex) operated under Analyst TF 1.5.1 control. Peptides were eluted at a constant flow of 250 nl/min with a 50 min gradient from 5 to 35 % solvent B (90 % ACN, 0.1 % formic acid) followed by re-equilibration for 10 min back to the starting conditions. Information dependent acquisition was employed acquiring up to 25 MS/MS spectra per cycle using 1.6 s cycle time with an exclusion window of 6 s.

### SILAC data analysis

All raw MS files were processed using Mascot Distiller 2.5.0 (Matrix Science). The MS data obtained by the analysis of gel lanes were merged into a multi-file-project using the default settings from the ABSciex_5600.opt file except that the MS/MS Peak Picking “Same as MS Peak Picking” was deselected and “Fit method” was set to “Single Peak”. After peak picking all scans, the data were searched against Swiss-Prot *Homo Sapiens* database (v. 2013_11/12) using Mascot v. 2.3.02 (Matrix Science) [67]. The Search parameters allowed one missed trypsin cleavage site, propionamide as a fixed modification, and oxidation of methionine as a variable modification. The mass accuracy of the precursor and product ions were set to 10 ppm and 0.2 Da, respectively, the instrument setting was specified as ESI-QUAD-TOF, and a significance threshold of 0.01 was used. The default SILAC R + 6 R + 10 [MD] quantitation protocol was selected using a significance threshold at 0.01, matched rho was 0.7, XIC threshold was 0.1, isolated precursor threshold was set at 0.5 and normalization set to median. Mascot Distiller results were exported to xml files, imported into MS Data Miner v.1.2.0 (http://sourceforge.net/p/msdataminer [68]) and exported into Excel reports. Keratin was excluded as a general contaminant and a fold regulation threshold of 1.3 was selected for significant differentially regulated proteins. Spectra for differentially regulated proteins were manually inspected and proteins were excluded if not at least two unique peptides showed peaks for both light, medium and heavy label significantly above background (ion counts > 50). The data are available via ProteomeXchange with identifier PXD000760 (http://proteomecentral.proteomexchange.org). Clustering analysis was done using the Functional Annotation tools of DAVID Bioinformatics Resources 6.7 [69, 70].

### Incubation of HepG2-SF cells in fatty acid supplemented media for DIGE

Four 75 cm^2^ culture flasks containing 90 % confluent cells were trypsinized and seeded into 16 new flasks (4 replicas/group), and allowed to attach for two days before addition of fatty acid supplemented medium (10 ml/flask). Medium was changed on day 3 and 6. On day 6 only 5 ml fatty acid supplemented medium was added. The 5 ml conditioned medium was collected on day 7, filtered, and stored on ice. Samples for DIGE was depleted for albumin as described for SILAC above and desalted using a PD10 column into 2.5 mM Tris, pH 7.4. Aliquots of 50 μg protein were dried in a SpeedVac and stored at −20 °C until use.

### DIGE analysis of proteins secreted by HepG2-SF cells

A total of 50 μg of protein was labeled using GE Healthcare CyDye DIGE Fluors (minimal dyes) according to the manufacturers instructions (GE Healthcare). The samples were labeled using 400 pmol Cy3 or Cy5 and as an internal standard, a pool of 25 μg of each sample to be analyzed was labeled by 400 pmol Cy2 per 50 μg protein. Labeling was conducted in 7 M Urea, 2 M Thiourea, 4 % w/v CHAPS, 10 mM Tris, pH 8.5. After 30 min incubation the labeling reaction was quenched by adding 1 μl 10 mM lysine. Four experimental groups with 4 replicas were analyzed (two replicas in each group labeled with Cy3 and two with Cy5) resulting in a total of 8 analytical DIGE gels. The combination of samples differed in all gels and no gels contained two replicas from the same experimental group. Two samples labeled with Cy3 and Cy5 respectively were mixed with 50 μg Cy2-labeled internal standard and volume adjusted to 130 μl in rehydration solution (7 M Urea, 2 M Thiourea, 4 % w/v CHAPS, 0.5 % v/v pH4-7 IPG carrier ampholytes, 40 mM DTT, 10 mM Tris, pH 8.5). The mixed samples were incubated in the dark, rotating at room temperature for 20 min and centrifuged 30 min at 16000 g. Supernatants were subjected to isoelectric focusing by cup-loading on 24 cm pH 4–7 Immobiline Drystrips (GE Healthcare) rehydrated overnight in rehydration solution. Isoelectric focusing was performed in an Ettan IPGphor II (GE Healthcare) at 25 °C with maximum 50 μAmp/strip using the following program: step 1 h at 150 V, gradient 1 h to 500 V, step 2 h at 500 V, gradient 7 h to 1000 V, gradient 4 h to 8000 V, step 5 h 8000 V.

After focusing, proteins in IPG-strips were reduced with DTT (10 mg/ml in equilibration buffer composed of 50 mM Tris, 6 M Urea, 30 % glycerol, 2 % SDS, pH 8.8) for 15 min followed by alkylation 15 min with iodoacetamide (25 mg/ml in equilibration buffer). The strips were equilibrated in upper reservoir Laemmli buffer (50 mM Tris, 384 mM Glycine, 0.2 % w/v SDS) and placed on top of an acrylamide/bis (10 % T, 0.87 % C) Tris–HCl, pH 8.8, gel in low fluorescent glass plates. Second dimension electrophoresis was conducted in the Ettan Dalt six gel system (GE Healthcare) program as follows: 2 h at 0.3 W/gel, 10 h at 1 W/gel, 4 h at 2 W/gel.

DIGE-gels were scanned on Typhoon 8600 scanner at a pixel resolution of 100 μm, the photomultiplier tubes at 600 V and laser and emission filters set at standard DIGE scanning. DeCyder software v. 6.0 (GE Healthcare) was used for image analysis. Spots more than 1.3 fold regulated with a *p*-value < 0.05 were selected for spot-picking.

A preparative gel with a protein load of 400 μg protein was prepared using the same conditions as described above. The gel was stained using coomassie blue G250 (Merck) and scanned (red laser, 633 nm). In DeCyder the gel was aligned with the DIGE gels and regulated spots were picked using an Ettan spotpicker (GE Healthcare).

### Identification of proteins of interest using LC MS/MS

Picked gel plugs were dehydrated and destained using acetonitrile and NH_4_HCO_3_ and the gel-plugs were dried before reswelling in 50 mM NH_4_HCO_3_ with 100 ng trypsin (Promega) pr plug and left over night at 37 °C for in-gel digestion [66]. At the end of digestion, formic acid was added. Peptides were purified on C18 reverse phase material using Stage-tips and eluted with 2 μl 70 % acetonitrile into 18 μl 0.1 % formic acid. The samples were subjected to LC MS/MS using an Easy nLC (Proxeon) (50 min gradient of 5–40 % acetonitrile in 0.1 % formic acid) coupled in-line with a Q-TOF Ultima API mass-spectrometer (Micromass/Waters). Data were processed using MassLynx 4.0 software (Waters). MS/MS data are searched against SwissProt (v. 2014_02) limited to human taxonomy using Mascot v. 2.3.02 (Matrix Sciences). Trypsin was selected and a maximum of one missed cleavage was allowed. The following variable modifications were accepted: Carbamidomethyl, propionamide, and oxidation on methionine. Peptide tolerance was set at 1.2 Da and MS/MS tolerance at 0.6 Da. Instrument setting was specified as ESI-QUAD-TOF. Protein identifications were based on significant MOWSE-score, peptide scores > 30, low e-values, and visual inspection of MS/MS spectra. Spots were excluded from further analysis if identified as albumin, due to the use of albumin as delivery vehicle for fatty acids.

## Additional files

Additional file 1: Data S1. GLC. Results from the gas–liquid chromatography analysis. On the left are in bold averages of mol% for three biological replicates and on the right their respective standard deviations. Raw data is provided for each replicate in separate sheets. (XLSX 48 kb) Additional file 2: Data S2. SILAC *trans*VA-EA- *cis*VA. Data from the SILAC investigation of the cellular response to *trans*VA, EA and *cis*VA. (XLSX 867 kb) Additional file 3: Data S3. DIGE identifications. Protein identifications by LC-MS/MS from DIGE analysis of the secretomes from cells incubated with *trans*VA, EA and *cis*VA. Spots with significantly different intensities between samples in the DIGE analysis were excised, and the proteins were identified by LC-MS/MS and Mascot searches. Proteins identified by ≥ 2 unique peptides with ion scores ≥ 30 are highlighted. (XLSX 23 kb) Additional file 4: Figure S1. DIGE 2D-gel with indications of the significant differentially regulated spots in EA/*trans*VA (panel A) and *trans*VA/*cis*VA (panel B). The numbers correspond to Table 2 and supplemental data S3. (DOCX 1198 kb) Additional file 5: Data S4. SILAC *trans*VA-OA-CLA. Data from the SILAC investigation of the cellular response to *trans*VA, OA and CLA. In sheet 1, “full data”, unfiltered data from this second SILAC triplex is displayed. In sheet 2, “signif. 1.3 fold”, proteins significantly regulated ≥ 1.3 fold in ≥ one comparison are displayed. Proteins are reported with Uniprot accession number, Name, Fold regulation in individual comparisons, the geometric standard deviation (Geo.SD.), and selected GO-terms: GO:0006950 response to stress (x) and GO:0002526 acute inflammatory response (y). Black bold: significant regulatory data (*p* < 0.01) with fold > 1.3, black italic: significant regulatory data (*p* < 0.01) with fold < 1.3, grey italic: not significant regulatory data (*p* > 0.01). (XLSX 925 kb)

## Abbreviations

CLA: Conjugated linoleic acid, (*trans,cis*∆^9,11^C18:2)
*cis*VA: cis-vaccenic acid (*cis*∆^11^C18:1)
CVD: Cardiovascular disease
DIGE: Difference in-gel electrophoresis
EA: Elaidic acid (*trans*∆^9^C18:1)
FAME: Fatty acid methyl esters
iTFA: Industrially produced TFA
OA: Oleic acid (*cis*∆^9^C18:1)
rTFA: Ruminant TFA
SILAC: Stable isotope labeling by amino acids in cell culture
TFA: Trans fatty acid
TG: triacylglycerol
*trans*VA: *trans*-vaccenic acid (*trans*∆^11^C18:1)
Protein name abbreviations: AHSG: alpha-2-HS-glycoprotein
CO3: complement factor C3
IGFBP1: insulin-like growth factor-binding protein 1
PAI1: plasminogen activator inhibitor 1
PCSK9: proprotein convertase subtilisin/kexin type 9
TFPI: tissue factor pathway inhibitor
TIMP1: metalloproteinase inhibitor 1
TTR: transthyretin
