# Supplementary material for: Hepatocytes respond differently to major dietary trans fatty acid isomers, elaidic acid and trans-vaccenic acid
Source: Proteome Sci. 2015 Dec 1;13:31. doi: 10.1186/s12953-015-0084-3 (PMC4665887; doi:10.1186/s12953-015-0084-3)
Supplement: Additional file 4: Figure S1. — DIGE 2D-gel with indications of the significant differentially regulated spots in EA/transVA (panel A) and transVA/cisVA (panel B). The numbers correspond to Table 2 and supplemental data S3. (DOCX 1198 kb) [file 12953_2015_84_MOESM4_ESM.docx]

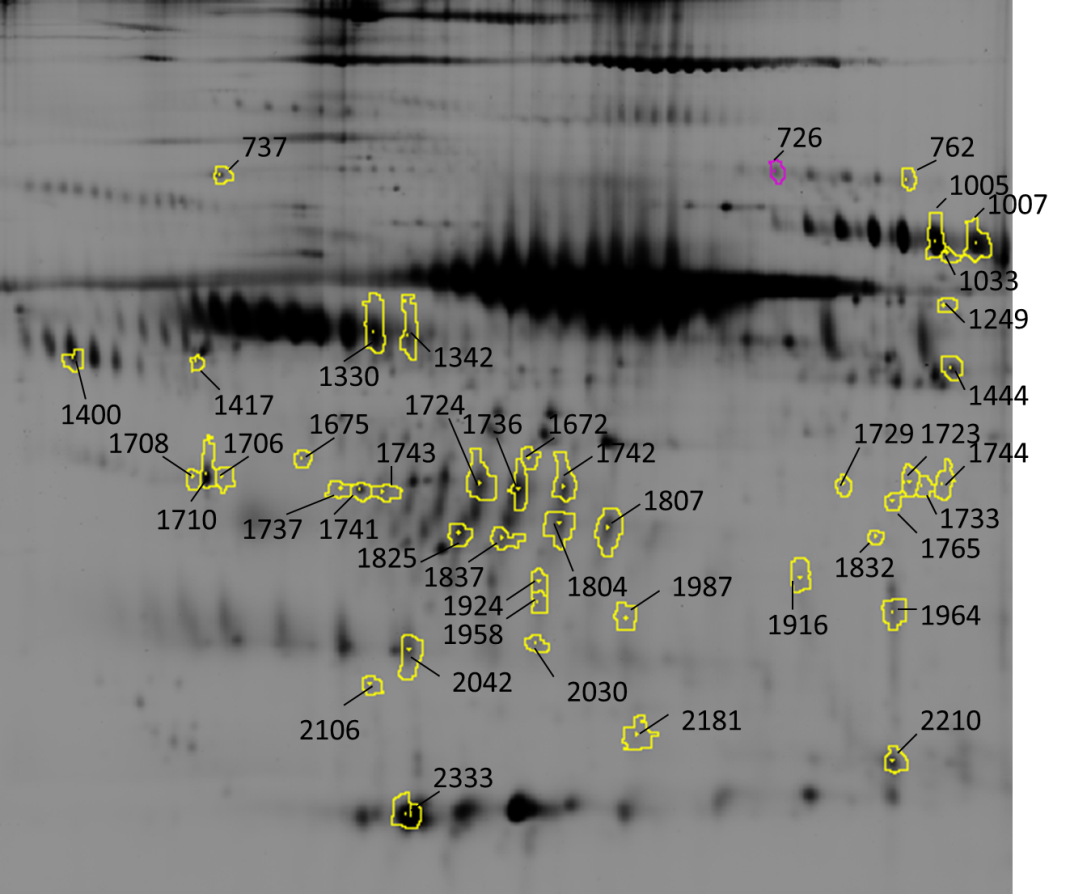
**Supplementary figure S1.** DIGE 2D-gel with indications of the significant differentially regulated spots in EA/VA (A) and VA/CVA (B). The numbers correspond to table 2 and supplementary data S3.

pH 7

pH 4

A: Spots differentially regulated between *Elaidic* and *trans-Vaccenic* with spot-numbers displayed


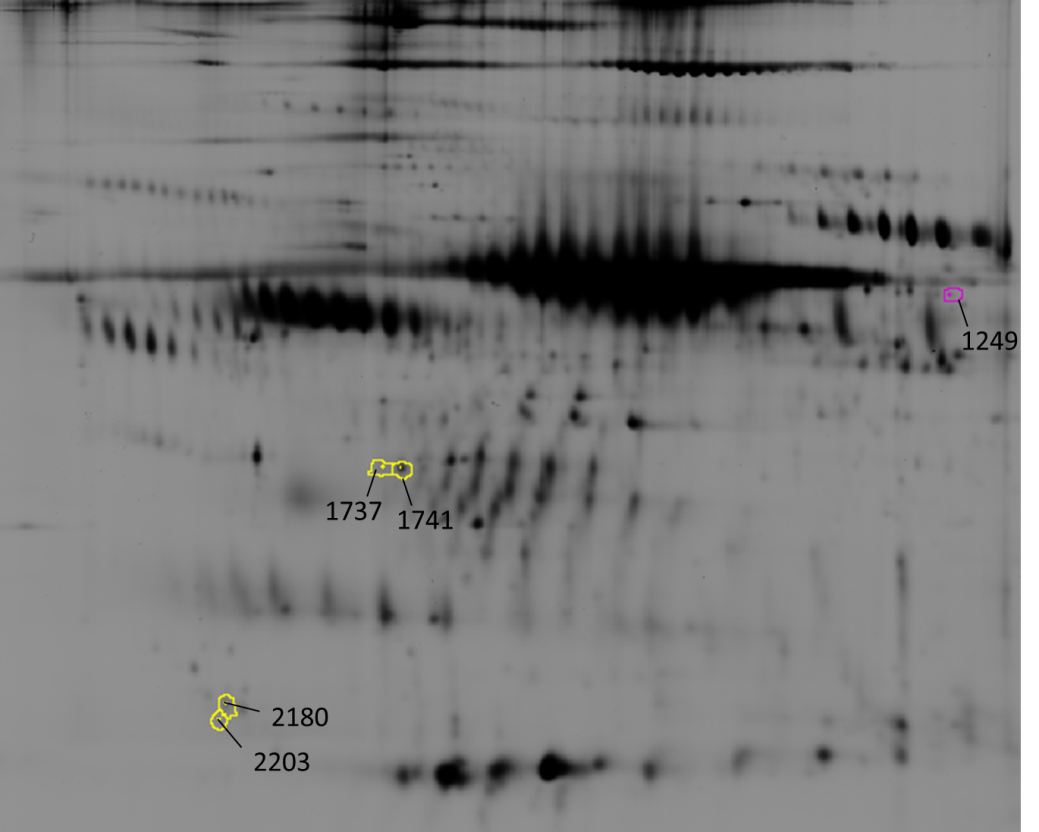


pH 7

pH 4

B: Spots differentially regulated between *trans-Vaccenic* and *cis-Vaccenic* with spot-numbers displayed
